# Assessing the immunogenicity and toxicity of the AFPL1-conjugate nicotine vaccine using heterologous and homologous vaccination routes

**DOI:** 10.1371/journal.pone.0221708

**Published:** 2019-08-23

**Authors:** Nya L. Fraleigh, Reynaldo Oliva, Jordan D. Lewicky, Alexandrine L. Martel, Reinaldo Acevedo, García-Rivera Dagmar, Hoang-Thanh Le

**Affiliations:** 1 Health Sciences North Research Institute, Sudbury, Ontario, Canada; 2 Finlay Institute of Vaccines, Havana, Cuba; 3 Northern Ontario School of Medicine, Medicinal Sciences division, Sudbury, Ontario, Canada; 4 Chemistry & Biochemistry and Biology Departments, Laurentian university, Sudbury, Ontario, Canada; University of Strathclyde, UNITED KINGDOM

## Abstract

Despite the increased risks of cancers and cardiovascular related diseases, tobacco smoking continues to be prevalent in the population due largely in part to the addictive nature of nicotine. Nicotine vaccines are an attractive alternative to the current smoking cessation options but have yet to be successful enough in clinical trials to reach the market due to a lack of neutralizing antibodies and inconsistent results. Using AFPL1 derived from the Cuban meningococcal vaccine as an adjuvant, we have previously published promising results with an intranasally administered nicotine vaccine. In order to examine the immunogenicity and safety of this vaccine in mice we set up a pilot trial administering the vaccine either intranasally, intramuscularly or utilizing both routes simultaneously and evaluated immune responses and clinical symptoms throughout the duration of the vaccination protocol and post-mortem. These data further demonstrate the ability of the AFPL1 nicotine conjugate vaccine to be a safe and potential candidate for clinical use.

## Introduction

Over the last 30 years the use of tobacco in Canada has decreased by approximately 20% [1]. Despite this, tobacco use continues to be a major contributor to increased risks of cancer and cardiovascular disease. Due to the addictive nature of nicotine, levels of success are moderate at best for people attempting to quit smoking tobacco when using traditional smoking cessation products and pharmacotherapeutics [2]. Immunotherapeutics, such as an anti-nicotine vaccine, present an interesting alternative to the current therapeutics that are available for smoking cessation. In theory, an anti-nicotine vaccine would direct the immune system to recognize nicotine, a hapten, and produce nicotine-specific antibodies that would bind to nicotine in the blood and prevent it from crossing the blood-brain barrier. Previous conjugate nicotine vaccines have been successful in preclinical evaluations but have provided limited success in clinical trials [3–6]. While a subpopulation of those that took the vaccine were able to respond due to high titers against nicotine [4], the overall consensus is that these vaccines, while promising, need stronger delivery systems that more effectively activate the immune system [5], which has led to the development of the next generation of nicotine vaccines in preclinical testing [6–11]. In addition, the delivery of nicotine to the brain occurs within 7–10 seconds of cigarette smoke inhalation [12], such that systemic antibodies alone may not be fast enough to neutralize absorbed nicotine and prevent it from reaching the brain. We believe that a successful nicotine vaccine needs to be able to generate both mucosal and systemic responses directed against nicotine.

With an intranasal (IN) administration strategy, the vaccine was delivered to the mucosal surfaces of the respiratory system. The anti-nicotine antibodies induced by the vaccine would theoretically be able to sequester nicotine using both systemic IgG, and mucosal IgA in the respiratory tract. We have previously published a novel intranasally delivered conjugate-nicotine vaccine that utilized the adjuvant Finlay proteoliposome 1 (AFPL1) as the adjuvant portion [13]. The vaccine demonstrated a significant ability to induce anti-nicotine antibodies that were able to prevent nicotine from reaching the brain upon an *in vivo* challenge with [H^3^]-nicotine [13]. [H^3^]-nicotine was found in equal amounts in the lung and the blood, likely due in part to both mucosal and systemic antibodies induced by the IN route. This would suggest value in having both mucosal and systemic antibodies, supplying two levels of protection in the lung and blood. In keeping with the goal of generating more readily available antibodies with a reduced number of vaccinations, we hypothesized that we could improve the ability of the AFPL1-conjugate nicotine vaccine by adopting a heterologous simultaneous vaccination at the priming event with two subsequent intranasal boosts. Heterologous simultaneous vaccination has been described using a variety of different routes and vaccines [14–17] with the focus to induce a stronger antibody response, especially in the mucosa, using fewer vaccination events [14].

Although AFPL1 has been used as part of the meningococcal vaccine in Cuba for decades and extensively researched [15, 18–24], it is still imperative that our nicotine vaccine be assessed in preclinical trials for not only its potency and immunogenicity but also for toxicity in both inbred and outbred rodent models [25]. This is especially true given that we are using a non-traditional intranasal route of administration. As a continuation of our previously published data, we evaluated whether there was toxicity associated with the AFPL1 conjugate nicotine vaccine in BALB/c mice and whether using a homologous or heterologous vaccination strategy would generate the best systemic and mucosal immune responses. To that end, we evaluated the vaccine delivered intramuscularly (IM) or IN as compared to combining the two routes of administration for the priming event with subsequent IN boosts. We assessed the immunogenicity of the vaccine both systemically and mucosally, determining whether the antibodies were able to bind to nicotine in an *in vivo* challenge model. The toxicity of the vaccine as compared to the delivery system alone and a negative control (PBS) was determined by measuring body weight, temperature, food and water consumption, in addition to local inflammation at the site of injection for the IM administered vaccines. Mice were physically monitored for conditions associated with stress and malaise including but not limited to physical symptoms such as changes in gait and posture and the condition of their fur. Post-mortem we evaluated each of the organs and in particular the spleen for immunotoxicity. The results demonstrate that the vaccine is most effective when delivered as part of the heterologous vaccination strategy and is safe when administered either IM, IN or as part of the heterologous vaccination protocol.

## Material & methods

### Animals and Husbandry

Female BALB/c mice were purchased from Charles River (QC, Canada) at an age of 6–8 weeks and were housed in Innocage^®^ mouse cages at the Animal Care Facility at Laurentian University. Mice were provided specialized feed for rodents, and the water used was provided in Aquavive^®^ acidified mouse water bottles (250 mL volume). Both food and water were available *ad libitum*. The animal room was maintained at a temperature of 21 ± 2 °C and a relative humidity of 55 ± 5%. These parameters were recorded daily in addition to maintaining 12 hour light and dark cycles. Mice were allowed to acclimatize to their surroundings for one week prior to the commencement of the experimental protocol and were randomly placed into groups of 5. All protocols were approved by the Animal Care Committee at Laurentian University and the Biosafety Committee at Health Sciences North Research Institute.

### Vaccines and vaccination protocol

The vaccine was prepared via the conjugation of 3’-aminomethylnicotine (Toronto Research Chemicals Inc., 25 mg/mL in MeOH) and the AFPL1 component (Finlay Institute of Vaccines, 2–5 mg/mL in water based on protein content determined using Thermo Scientific Pierce BCA Protein Assay) at pH 5–6 in the presence of EDC coupling reagent (Sigma Aldrich, 10 equiv. based on nicotine hapten concentration) and a synthetic matrix peptide developed in our lab [13] (maximizes the coupling efficacy of the nicotine hapten). During the conjugation reaction, nicotine was quantified using UV absorption and TLC on silica gel 60 F254 with detection by Dragendorff reagent staining. The final conjugation product was purified by dialysis in HEPES buffer with 0.01% Tween 80, and then lyophilized using different freeze drying techniques. Particle size and zeta potential were characterized using a Malven Zetasizer ZS. The conjugate-nicotine vaccine (2–5 mg/mL based on nicotine concentration) was stored at either 4°C for one year (solution form) or at room temperature (solid form).

Mice were immunized by one of three methods (Table 1): two single routes of administration included either IN or IM for a total of three immunizations per group, while the other vaccination group was administered a simultaneous combination of IN/IM as the first dose and the remaining doses were administered IN until the three doses were completed. The vaccine was administered once every three weeks at a dose of 10 μg per route, based on the nicotine concentration, in different volumes such that that the IN vaccination volume was 20 μL (10 μL for each nare) and the IM vaccination volume was 100 μL (50 μL per leg). Sera was collected by retro-orbital bleeds two weeks post-vaccination and mice were sacrificed at either 21 day after the final vaccination or 27 days after the final vaccination when challenged with nicotine.

**Table 1:** Vaccine experimental design.

Group	Route	Animals, *n*	*n* Euthanized at x Days After 3^rd^ Dose	
			*x = 21*	*x = 27*
PBS	IM	5	5	-
	IN	5	5	-
	IM/IN	5	5	-
Adjuvant (AFPL1)	IM	4	-	-
	IN	5	-	-
	IM/IN	5	-	5
Vaccine	IM	5	5	-
	IN	5	5	-
	IM/IN	10	5	5

### Clinical observations and symptoms

All observations started from the experimental zero time (T_0_), which was considered as the same day as the first vaccination event. Animals were monitored daily with extra attention being paid to the mice when their food, water, weight, leg muscle diameter and temperature were being measured. Weight, food and water consumption were measured weekly (g or mL or g/animal/day respectively) as measures of toxicity.

The body temperature of the mice was measured with a laser clinical thermometer (Equate^®^, non-contact forehead thermometer, model # 10857) directed towards the right ear. Body temperature was measured before each inoculation and 24 hours after. In the event of observing an increase in temperature that would indicate a fever, the measurements would have continued until the body temperature returned to normal.

The evaluation of the muscle diameter was performed as previously described for rats [25] with a digital caliper (Mastercraft, Electronic Caliper with digital display, 6”, 150 mm) by measuring the diameter of the inoculated limb before the IM vaccination. Briefly, the mouse was restrained and the measurement of muscle diameter was taken at the center of the musculature of the thigh region. Leaving the teeth of the caliper on the inner and external side of the muscle, the caliper was closed without putting pressure on the leg. This was done before each IM injection and 24 hours after to assess local inflammation.

### Euthanasia, blood collection and bronchoalveolar lavages

Mice were sacrificed under excess isoflurane followed by a cardiac puncture and cutting the diaphragm either 21 or 27 days after the final vaccination. Blood was collected by a cardiac puncture and placed in sera tubes which were spun at 10 000 rpm for 5 minutes. Sera was aliquoted and frozen for later analysis. A bronchoalveolar lavage (BAL) was performed by cannulating the trachea, instilling 0.5 mL of PBS into the lung and aspirating it back. The samples were pelleted to generate cell-free BAL for ELISA analysis.

### Anatomopathological studies and organ weights

The anatomopathological studies for necropsy (macroscopic evaluation) were performed immediately after euthanasia. All organs and sites of vaccine administration were examined macroscopically. Solid or parenchymal organs (brain, heart, lungs, spleen, liver and kidneys) and thymus were removed and weights were recorded. They are expressed as relative weight (RW), and were calculated by the following equation:

RW=(OWx100)/EEW (1)

where OW is the organ weight, and EEW is the animal end weight on the day of euthanasia.

### Immunotoxicological evaluation

Systemic inflammation was assessed by using the ImagJ software [26] and measuring the total area of each of the spleens of the animals by groups. The results obtained were compared between all of the vaccinated groups.

### Immunological evaluation

Sera and BAL were analyzed by an indirect ELISA as previously described [13]. Biotinylated goat anti-mouse IgG and IgA were used as secondary antibodies to detect the nicotine-specific systemic and mucosal antibodies found in the sera and BAL. The plates were incubation with p-Nitrophenyl phosphate (pNPP, ≥97%, Sigma Aldrich) and the reaction was stopped with 3N NaOH after 30 minutes. The plates were read at an OD of 405 nm with a subtraction of 490 nm.

### *In vivo* intranasal nicotine challenge

On day 27 after the final vaccination, mice were anaesthetized with isoflurane before instilling intranasally a 0.03 mg/kg dose of nicotine (solution of neutralized (-)-nicotine hydrogen tartrate salt (≥98%, Sigma Aldrich) in PBS, 10 μL per nare) which is the equivalent of 1–2 cigarettes [27]. The mouse was kept under isoflurane for 5 minutes before sacrificing using a cardiac puncture to remove 700 μL of blood and cutting the diaphragm while under excess isoflurane. Post-mortem BALs were collected for ELISAs that were immediately performed after collecting the samples. Samples were compared to similar samples collected from the non-nicotine challenged mice that were euthanized one week prior.

### Statistical analysis

Statistical analyses were performed using Graph Pad Prism 5. Multiwise group analyses were performed using either a nonparametric ANOVA with a Kruskal-Wallis and a Dunn’s post-hoc test or an ANOVA with a Tukey HSD. Data were considered significant when p ≤ 0.05.

## Results

### Toxicity assessments

**Fig 1:**
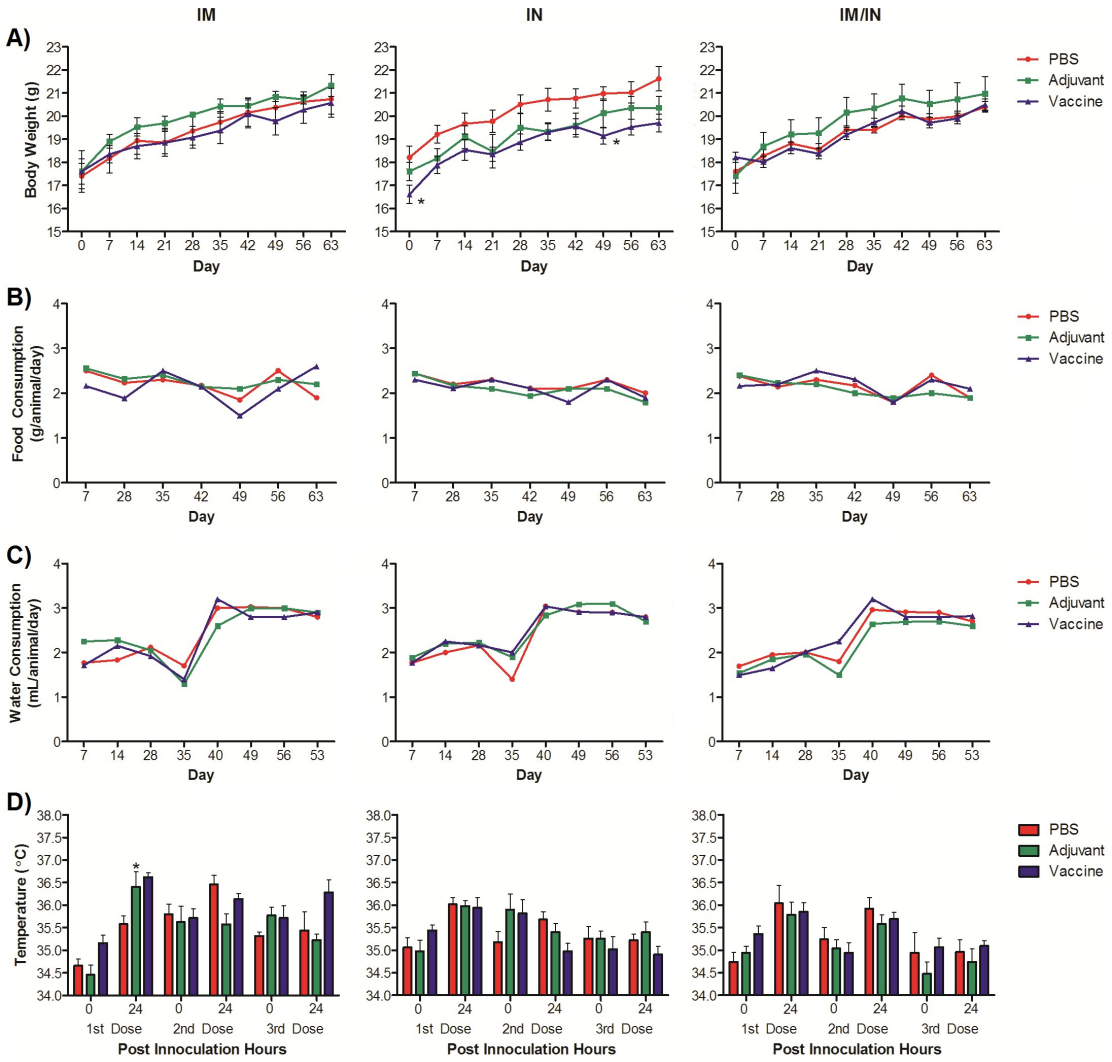
Clinical observations of mice vaccinated with the nicotine vaccine candidate via homologous and heterologous routes. A) Mice were weighed throughout the vaccination protocol. Each value represents the average ± SEM of all animals in each group. * p ≤ 0.05 as compared to PBS group at same time point. B) & C) Food and water consumption were monitored throughout the vaccination protocol. Each value represents consumption relative to all animals in the groups. D) Corporal temperatures of the mice were measured before and 24 hours after each vaccination. Each value represents the average ± SEM of all animals in each group. * p ≤ 0.05 as compared to the pre-vaccination measurement.

We vaccinated mice using our previously published conjugate AFPL1 nicotine vaccine using different administration routes to assess toxicity at the end of the vaccination protocol. No mortality or abnormal clinical signs were noted during the study. All of the female BALB/c mice increased their body weight during the 63 days of the study (Fig 1A). The weight increase curves of the mice were similar to those observed for this species and in line with the growth curves available from Charles River. However, a statistical difference was observed at day zero (before receiving the vaccine) and day 49 for the group receiving the IN nicotine vaccine as compared to the control groups but were back on par with their control a week later.

We also evaluated the amount of food (Fig 1B) and water (Fig 1C) consumed by the mice over the span of the vaccination protocol. Both followed the same trends between each group for the routes of administration while the mice continued to gain weight.

The normal physiological body temperature range reported for mice is 36.5 to 38.0 °C [28, 29]. The mice in this study had no increase in body temperature that could be considered a fever (Fig 1D). This lack of fever was reported in all groups including the mice that were vaccinated with the heterologous simultaneous vaccination protocol. The average temperature of the mice before each dose was 35.6 ± 0.6 °C and 24 hours later was 35.7 ± 0.6 °C, which was lower than normal and was consistent throughout the study. This may have been due to a setting on the thermometer or ambient room conditions and not reflective of the mice being in distress. There was a significant increase in temperature 24 hours after the first IM adjuvant dose as compared to the other routes of administration (p ≤ 0.05), although it was not clinically relevant as it did not rise above 38 °C.

In order to assess the inflammation induced by the vaccine administered intramuscularly, the muscle diameter of the legs was measured before and after receiving the IM vaccination. No significant differences were observed between the groups (Table 2) 24 hours after each dose. However, animals vaccinated by the IM route showed a tendency to have a larger muscle diameter than the AFPL1 and PBS groups after the third dose (Table 2).

**Table 2:** Muscle diameter measurements of mice vaccinated with the nicotine vaccine candidate via homologous and heterologous routes. The muscle diameters of both hind legs were measure for each mouse before and 24 hours after each vaccination. Each value represents the average ± SEM of both legs for all animals in each group.

		Dose #1		Dose #2		Dose #3	
		*0 Hours*	*24 Hours*	*0 Hours*	*24 Hours*	*0 Hours*	*24 Hours*
**PBS**	IM	4.11 ± 0.07	4.25 ± 0.08	4.08 ± 0.05	4.13 ± 0.03	4.07 ± 0.03	4.06 ± 0.01
	IM/IN	4.12 ± 0.05	4.13 ± 0.03	-	-	-	-
**Adjuvant**	IM	4.21 ± 0.06	4.27 ± 0.04	4.13 ± 0.03	4.08 ± 0.03	4.07 ± 0.03	4.06 ± 0.02
	IM/IN	4.15 ± 0.03	4.15 ± 0.03	-	-	-	-
**Vaccine**	IM	4.20 ± 0.05	4.23 ± 0.04	4.08 ± 0.01	4.10 ± 0.02	4.05 ± 0.02	4.13 ± 0.08
	IM/IN	4.23 ± 0.03	4.17 ± 0.02	-	-	-	-

Macroscopic studies performed on all organs and systems for each of the mice studied did not show any lesions suggesting acute or chronic toxicity. Administration sites showed no perceptible local changes. Regarding the relative organ weights (Table 3), no significant differences were observed for any organ between the vaccination groups, regardless of the administration route. A joint analysis of the relative weights of the organs, as well as the body weights of the animals in the study, led us to conclude that these variables were not affected by the route or the vaccine itself. The spleens were further analyzed to determine whether chronic systemic inflammation was present. The macroscopic morphometric evaluation of the spleen diameter for each of the animals of the different groups did not show significant differences in the total areas of this organ (Table 4). This evaluation gives us a preliminary view of possible immunotoxicological effects, given that neither the relative weight of the spleens, nor the total area of this organ evaluated by macroscopic morphometry showed statistical differences.

**Table 3:** Relative organ weights (%) of mice vaccinated with candidate nicotine vaccine. Each value represents the average ± SD of the 5 animals in the groups.

Group	Relative Organ Weights (%)								
	Brain	Thymus	Heart	Left Lung	Right Lung	Liver	Spleen	Left Kidney	Right Kidney
	*p = 0*.*3125*	*p = 0*.*8125*	*p = 0*.*4375*	*p = 0*.*6250*	*p = 0*.*6845*	*p = 0*.*8125*	*p = 0*.*0625*	*p = 0*.*6250*	*p = 0*.*4375*
IM PBS	2.144±0.04	0.157±0.02	0.386±0.01	0.354±0.08	0.501±0.06	4.456±0.21	0.451±0.03	0.525±0.03	0.531±0.04
IM Vaccine	2.206±0.12	0.182±0.03	0.448±0.03	0.408±0.06	0.509±0.10	4.688±0.28	0.425±0.03	0.608±0.03	0.598±0.02
	*p = 0*.*0625*	*p = 0*.*8125*	*p = 0*.*1875*	*p = 0*.*4375*	*p = 0*.*4375*	*p = 0*.*6250*	*p = 0*.*1875*	*p = 0*.*0625*	*p = 0*.*0625*
IN PBS	1.957±0.07	0.178±0.03	0.445±0.02	0.410±0.04	0.686±0.11	4.695±0.14	0.437±0.03	0.574±0.03	0.591±0.03
IN Vaccine	2.114±0.04	0.168±0.03	0.483±0.04	0.441±0.05	0.571±0.12	4.797±0.20	0.470±0.02	0.624±0.01	0.649±0.03
	*p = 0*.*8125*	*p = 0*.*0579*	*p = 0*.*6250*	*p = 0*.*6250*	*p = 0*.*1250*	*p = 0*.*6250*	*p = 0*.*3125*	*p = 0*.*4375*	*p = 0*.*3125*
IM/IN PBS	2.088±0.14	0.146±0.01	0.423±0.02	0.411±0.03	0.516±0.02	4.607±0.24	0.449±0.03	0.581±0.01	0.568±0.02
IM/IN Vaccine	2.029±0.10	0.174±0.01	0.441±0.03	0.395±0.06	0.600±0.06	4.754±0.20	0.473±0.03	0.613±0.06	0.616±0.04
	*p = 0*.*4375*	*p = 1*.*0000*	*p = 0*.*1250*	*p = 0*.*0625*	*p = 0*.*8125*	*p = 0*.*0625*	*p = 0*.*2785*	*p = 0*.*3125*	*p = 0*.*3125*
IM/IN Adjuvant	1.991±0.12	0.165±0.03	0.432±0.03	0.327±0.03	0.526±0.11	4.397±0.16	0.418±0.01	0.559±0.02	0.563±0.03
IM/IN Vaccine	1.929±0.04	0.165±0.03	0.446±0.02	0.349±0.05	0.530±0.08	4.850±0.09	0.0445±0.04	0.592±0.04	0.604±0.03

**Table 4:** Morphometric evaluation of total macroscopic area of spleens of mice vaccinated with candidate nicotine vaccine. Values represent the average ± SD of the 5 animals in the groups.

Route	Area (cm^2^)			Perimeter (cm)		
	*Average*	*Min*	*Max*	*Average*	*Min*	*Max*
IN—PBS	0.700 ± 0.048	0.653	0.768	3.896 ± 0.157	3.721	4.129
IN—Vaccine	0.705 ± 0.044	0.654	0.766	3.827 ± 0.174	3.629	4.055
IM—PBS	0.737 ± 0.048	0.684	0.809	3.912 ± 0.254	3.664	4.339
IM—Vaccine	0.671 ± 0.025	0.636	0.689	3.849 ± 0.103	3.735	3.946
IM/IN—PBS	0.689 ± 0.050	0.625	0.758	3.863 ± 0.202	3.632	4.092
IM/IN—Vaccine	0.727 ± 0.082	0.642	0.833	3.939 ± 0.303	3.592	4.066
IM/IN—Adjuvant	0.712 ± 0.054	0.648	0.794	3.926 ± 0.216	3.810	4.263
IM/IN—Vaccine	0.712 ± 0.052	0.666	0.770	3.793 ± 0.105	3.652	3.906

### Immunological evaluations

**Fig 2:**
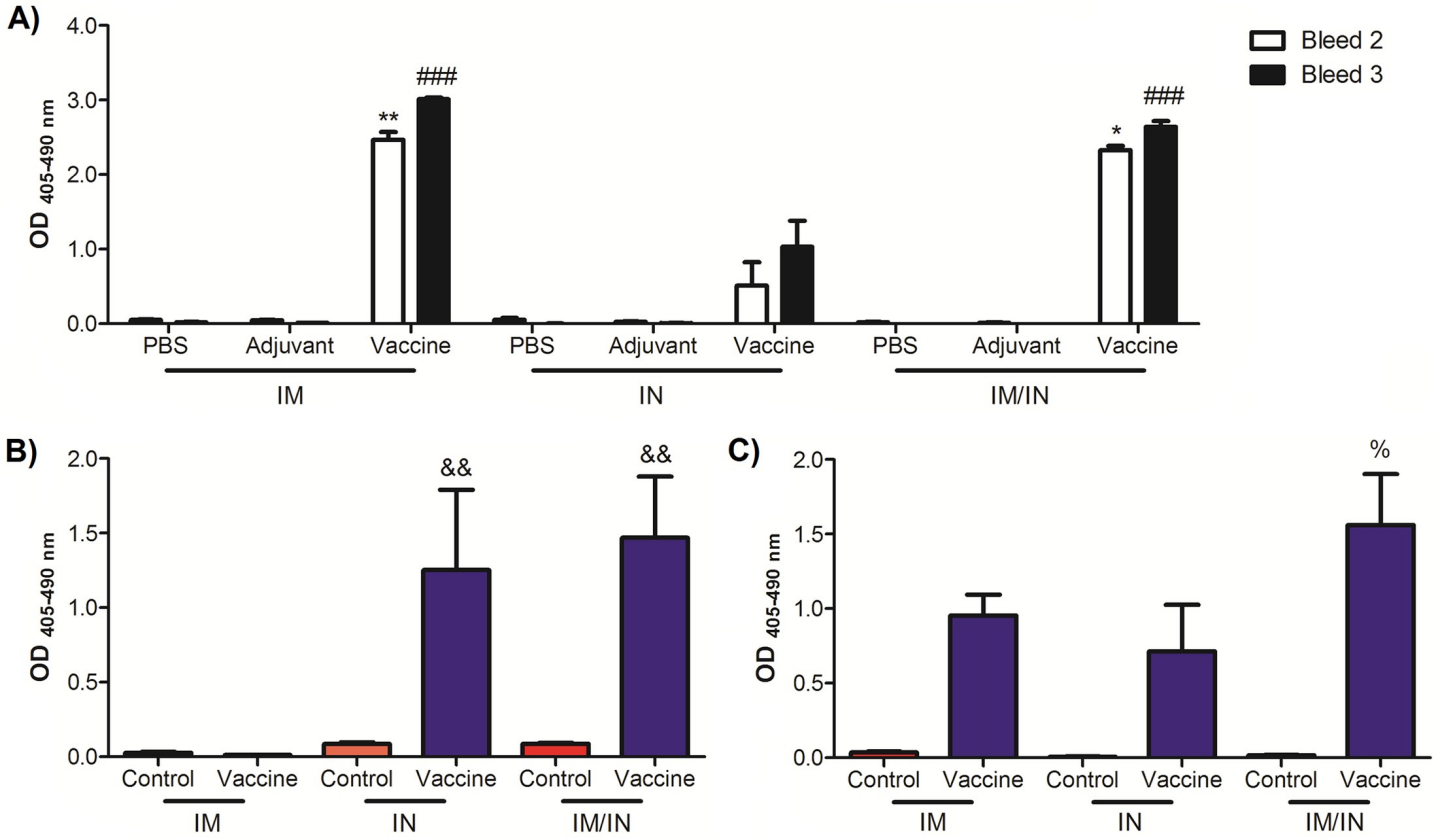
Anti-nicotine antibody responses in BALB/c mice vaccinated either by homologous or heterologous routes. Mice were vaccinated on days 0, 21, and 42 using the homologous (IM or IN) or heterologous (IM/IN) routes with either the controls or the nicotine vaccine. A) Sera IgG levels. On day 28 and 49 the mice were bled by retro-orbital bleed and sera was analyzed by an indirect ELISA for levels of anti-nicotine IgG. Sera was diluted 1:2400 and data are represented as ±SEM with 4–10 mice per group/bleed. Statistical significance for bleed 2 was determined by a Kruskal-Wallis test with a Dunn’s multiwise comparison, *p ≤ 0.05 and **p ≤ 0.01 as compared to the respective IN vaccine group. Statistical significance for bleed 3 was determined by an ANOVA with a Tukey HSD, ###p ≤ 0.001 as compared to the respective IN vaccine group. Bronchoalveolar lavages were collected at the end of the experimental protocol and B) levels of anti-nicotine IgA and C) IgG were determined by an indirect ELISA. Data are represented as ±SEM of each group of 4–5 mice. Statistical significant for B) and C) was determined by a Kruskal-Wallis test with a Dunn’s multiwise comparison, &&p ≤ 0.01 as compared to the IM vaccine group and %p ≤ 0.05 as compared to respective control.

Sera was collected after the first, second and third vaccination to assess systemic anti-nicotine IgG responses (only the second and third bleeds are shown). By the second vaccination there is already a clear difference between the amount of antibodies being produced by the heterologous and the homologous vaccination protocols. Multiple consecutive IM vaccinations yielded the stronger systemic responses by the third bleed (Fig 2A) while the heterologous vaccination protocol was stronger than the homologous IN protocol but not significantly different from the homologous IM vaccine group. There is also very little background OD from the sera of the negative control groups, demonstrating that the indirect ELISA protocol and antibodies produced by the vaccine are specifically directed against nicotine. Because we had vaccinated the mice using a mucosal route (IN), we evaluated the relative anti-nicotine response in the BALs from mice vaccinated by each route. Both the IN and the IM/IN vaccinated mice were able to produce anti-nicotine IgA in the lung which was significantly higher than the IM vaccinated group (Fig 2B). There was no significant difference in IgA response between the IN and the IM/IN groups. Similar levels of anti-nicotine IgG were found in all vaccinated groups but was only significantly higher than the control for the mice that were heterologously vaccinated (Fig 2C).

**Fig 3:**
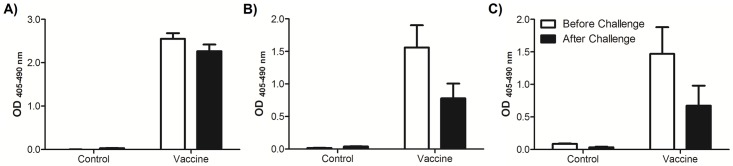
Levels of systemic and mucosal anti-nicotine antibodies before and after challenge. Mice vaccinated using the heterologous vaccination strategy were challenged with 0.03 mg/kg nicotine (solution of neutralized (-)-nicotine hydrogen tartrate salt in PBS) intranasally and 5 minutes later were sacrificed. Sera and BALs were collected and immediately analyzed by an indirect ELISA alongside similar samples from non-challenged mice. A) levels of anti-nicotine IgG in the sera of control and vaccinated mice, B) levels of anti-nicotine IgG and C) IgA in the BALs of control and vaccinated mice before and after the nicotine challenge.

To demonstrate that the antibodies generated by the vaccine could bind to nicotine, we performed an *in vivo* IN nicotine challenge. The heterologous vaccination groups were challenged intranasally with 0.03 mg/kg nicotine and sacrificed after 5 minutes to determine systemic (Fig 3A) and mucosal (Fig 3B and 3C) levels of anti-nicotine antibodies as compared to their non-challenged counterparts. The indirect ELISA would act as a competitive ELISA as the antibodies bound to nicotine would not have the ability to also bind to the plate. As compared to the non-challenged groups the level of anti-nicotine IgG decreased in the sera but remained relatively high in the lungs, with about 50% of both anti-nicotine IgA and IgG lost upon challenge.

## Discussion

Nicotine addiction remains a global health concern and the current smoking cessation products available have limitations for long term success. A nicotine vaccine is an attractive alternative but needs to be safe for the consumer while still being able to induce high levels of nicotine-specific antibodies. We have previously demonstrated that an intranasally delivered AFPL1-conjugate nicotine vaccine was able to induce significant levels of anti-nicotine antibodies that are able to block nicotine from crossing the blood brain barrier in [H^3^]-nicotine challenges [13]. Here we attempted to use simultaneous heterologous and homologous vaccination protocols to assess the immune responses generated by the different routes and the potential toxic effects of repeated administration of our conjugate nicotine vaccine. This repeated dose protocol not only maximizes the antibody production but also allows us to determine whether there are potential adverse side effects associated with the vaccine or the delivery system.

The mice were vaccinated either using the heterologous vaccination protocol with both IN and IM routes during the initial immunization event and two subsequent IN doses 3 weeks apart, or a homologous vaccination protocol comprising of either three IN or IM inoculations once every three weeks. All routes of administration generated anti-nicotine antibodies by the second bleed which increased after the final vaccination. While the homologous routes alone each show promise by inducing strong systemic (IM) or suitable systemic with the advantage of mucosally induced antibodies (IN); by adopting the heterologous strategy we get the combined advantages of both the strong systemic antibodies as seen with the IM alone and the mucosally derived antibodies that are induced from the IN administration, yielding the best results. Upon first glance this is not obvious when evaluating the sera alone; as expected the IM route and the heterologous IM/IN route generated significant levels of anti-nicotine IgG as compared to the IN group with no significant differences between the IM and IM/IN route. However, the IM vaccine was not able to induce mucosal anti-nicotine IgA in the BAL unlike both the IN and IM/IN routes. In addition, the variability in the mucosal responses is decreased with the heterologous administration route, and results in significant levels of IgA and IgG in the lungs, that which was not the case for either of the homologous routes. The additive effect from combining the IM and IN routes suggests that the IgG in the lung of the heterologously vaccinated mice was both mucosally derived and leaking from the systemic into the mucosa as it is known to be able to cross into the mucosal tissues [30]. It is not possible to directly compare results between research groups, given that the indirect ELISA protocols utilize different coatings, concentrations and methods. However, when comparing to other nicotine vaccines, the new nicotine vaccine system is able to generate both mucosal and systemic responses directed against nicotine.

To demonstrate that the anti-nicotine antibodies generated were able to neutralize nicotine, half of the heterologously IM/IN vaccinated mice were challenged IN *in vivo* with nicotine after the final vaccination. Indirect ELISAs were performed immediately post-mortem using the sera and the BALs collected from the control and vaccine groups in order to ensure that the nicotine-antibody complex was not compromised. As expected, the mice vaccinated with the nicotine vaccine were able to bind some of the nicotine *in vivo* which resulted in decreased levels of detection in the ELISA for antibodies in the sera and in the lung. Antibodies in the lung are readily available for first contact with nicotine, which may have reduced the burden of the antibodies in the sera to bind and neutralize nicotine during challenge. This information supports our hypothesis that the lung is an important target for a nicotine vaccine design.

Clinically, the mice from all of the groups displayed appropriate gains in weight, no anorexia or changes in food and water consumption over the period of the vaccination protocol. Body weight has been commonly considered in a wide range of toxicological studies as a sensitive and general indicator of the toxicity of xenobiotics [31–34]. The mice were weighed before the study began and were in the range of 16–20 g, and averaged 17.97 ± 0.93 g. We did notice that the IN group was significantly smaller at day 0 than the other groups of mice. These results were not due to the vaccine itself but just random chance of smaller mice being placed in one group or another since this time point for weight was prior to any vaccination events. The IN vaccine group’s weight was also significantly lower at day 49 but a week later was on par with the other groups. We do not believe this is a cause for concern as the other mice in the IM/IN vaccine group (n = 10) received the same IN doses and showed no differences at any time point as compared to the control groups. In addition, the average weight of each of the groups is within the range reported for the growth curve for the species by Charles River.

The evaluation of body temperature and local inflammation at the injection site for the IM route are parameters considered predictive for clinical trials [31]. In addition, these are evaluated in clinical trials of reactogenicity of vaccines [34], and this vaccine candidate can be considered of low reactogenicity. When a fever (>38 °C) is induced by some vaccines by intramuscular injection it is registered within the first 24 hours [35]. We measured the temperature of the mice before and after each vaccination to determine whether a fever was induced. There were significant differences recorded in regards to the IM vaccinated group, however this would lack clinical significance since the temperatures were still below the threshold for a fever. IM vaccines can have a longer time frame between vaccinations and the boosts are generally four weeks for more apart. However, here the change in temperature was not due to the shortened interval in between the vaccination dates as the heterologous vaccine group, which contained an initial IM dose, did not demonstrate an increase in basal temperature.

An important measure of whether a vaccine delivery system or the vaccine itself could be toxic is to systemically assess the immunotoxicity and the condition of the organs at a macroscopic level. This is especially relevant when delivering a vaccine IN as the nasal passages of rodents contain an olfactory bulb that can allow for endotoxins to have direct access the CNS resulting in systemic inflammation [36]. AFPL1 from the Finlay Institute is able to activate cells of the immune system through TLR2, 4- and 9, with TLR4 activation being due to trace amounts of LPS present in the proteoliposome extraction from *N*. *meningitidis* [15]. Toxicity associated with IN administration is usually seen behaviorally and would have yielded differences in food and water consumption [37] and eventual anorexia, which was not seen with our vaccine. There were no significant differences between the control groups and the vaccinated groups with respect to the weights of the lungs suggesting that there is no obvious lung changes because of the vaccine. This would need to be confirmed later with histopathological analyses of the organs. Systemic toxicity and immunotoxicity was assessed using the weights of the organs and area of the spleens, respectively, for each of the vaccination groups and administration routes used. No significant differences were observed for any of the groups or routes assessed in this study suggesting that the vaccine does not induce systemic toxicity or immunotoxicity.

Together, the data suggests that not only is the nicotine AFPL1 conjugate a good candidate as a nicotine vaccine based on our previous data in regards to its ability to block nicotine from crossing the blood brain barrier, but it is also non-toxic in a female BALB/c model. Additionally, we can significantly ameliorate the amount of both systemic and mucosal antibodies directed against nicotine by using the heterologous vaccination strategy which induces the same amount of systemic antibodies as compared to the traditional IM route while at the same time being able to produce the same or better mucosal anti-nicotine antibody response when compared to the IN route. Under the conditions of the study and the established criteria, the AFPL1-conjugate nicotine vaccine is immunogenic and potentially non-toxic.

## References

[1] Canadian Tobacco Use Monitoring Survey. 2013. https://www.canada.ca/en/health-canada/services/publications/healthy-living/canadian-tobacco-use-monitoring-survey-ctums-2012.html

[2] Moreno AY, Janda KD. Immunopharmacotherapy: vaccination strategies as a treatment for drug abuse and dependence. Pharmacol Biochem Behav. 2009; 92(2): 199–205. 10.1016/j.pbb.2009.01.015 19350728 PMC2768391

[3] Hatsukami DK, Rennard S, Jorenby D, Fiore M, Koopmeiners J, de Vos A, Hormith, et al Safety and immunogenicity of a nicotine conjugate vaccine in current smokers. Clin Pharmacol Ther. 2005; 78(5): 456–67. 10.1016/j.clpt.2005.08.007 16321612

[4] Vanschayck O. Nicotine vaccination—does it have a future? Addiction. 2014; 109(8): 1223–5. 10.1111/add.12569 24894565

[5] Pentel PR, LeSage MG. New directions in nicotine design and use. Adv Pharmacol. 2014; 69: 553–580. 10.1016/B978-0-12-420118-7.00014-7 24484987 PMC4047682

[6] Hoogsteder PHJ, Kotz D, van Spiegel PI, Viechtbauer W, van Schayck OC. Efficacy of the nicotine vaccine 3’-AmNic-rEPA (NicVAX) co-administered with varenicline and counselling for smoking cessation: a randomized placebo-controlled trial. Addiction. 2014; 109(8): 1252–59. 10.1111/add.12573 24894625

[7] Thorn JM, Bhattacharya K, Crutcher R, Sperry J, Isele C, Kelly B, et al The effect of physiochemical modification on the function of antibodies induced by anti-nicotine in mice. Vaccines. 2017; 5: E11 10.3390/vaccines5020011 28513561 PMC5492008

[8] Zhao Z, Harris B, Hu Y, Harmon T, Pentel PR, Ehrich M, et al Rational incorporation of molecular adjuvants into a hybrid nanoparticle-based nicotine vaccine for immunotherapy against nicotine addiction. Biomaterials. 2018; 155: 165–75. 10.1016/j.biomaterials.2017.11.021 29179132 PMC5738287

[9] Hu Y, Smith D, Frazier E, Hoerle R, Zhang C. The next-generation nicotine vaccine: a novel and potent hybrid nanoparticle based nicotine vaccine. Biomaterials. 2016; 106: 228–39. 10.1016/j.biomaterials.2016.08.028 27569868 PMC5018466

[10] McCluskie MJ, Thorn J, Gervais DP, Stead DR, Zhang N, Benoit M, et al Anti-nicotine vaccines: comparison of adjuvanted CRM197 and Qb-VLP conjugate formulations for immunogenicity and function in non-human primates. Int Immunopharamacol 2015; 29(2): 663–71. 10.1016/j.intimp.2015.09.012 26404190

[11] Zeigler DF, Roque R, Clegg CH. Optimization of a multivalent peptide vaccine for nicotine addiction. Vaccine 2019; 37; 1584–90. 10.1016/j.vaccine.2019.02.003 30772068 PMC6417836

[12] Quinn DI, Wodak A, Day RO. Pharmacokinetic and pharmacodynamics principles of illicit drug use and treatment of illicit drug users. Clin. Pharmacokinet. 1997; 33: 344–400. 10.2165/00003088-199733050-00003 9391747

[13] Fraleigh NL, Boudreau J, Bhardwaj N, Eng NF, Murad Y, Lafrenie R, et al Evaluating the immunogenicity of an intranasal vaccine against nicotine in mice using the Adjuvant Finlay Proteoliposome (AFPL1). Heliyon. 2016; 2(8): e00147 10.1016/j.heliyon.2016.e00147 27622215 PMC5008958

[14] Wern JE, Sorensen MR, Olsen AW, Andersen P, Follmann F. Simultaneous subcutaneous and intranasal administration of a CAF01-adjuvanted Chlamydia vaccine elicits elevated IgA and protective Th1/Th17 responses in the genital tract. Front Immunol. 2017; 8: 569 10.3389/fimmu.2017.00569 28567043 PMC5434101

[15] Pérez O, Romeu B, Cabrera O, González E, Batista-Duharte A, Labrada A, et al Adjuvants are key factors for the development of future vaccines: lessons from the Finlay adjuvant program. Front Immunol. 2013; 4:407 10.3389/fimmu.2013.00407 24348475 PMC3845353

[16] Gonzalez AE, Romeu B, Lastre M, Zayas C, Cuello M, Cabrera O, et al Mucosal and systemic immune responses induced by a single time vaccination strategy in mice. Can J Microbiol. 2015; 61(8): 531.8 10.1139/cjm-2015-0063 26140382

[17] Lorenzen E, Follmann F, Bøje S, Erneholm K, Olsen AW, Agerholm JS, et al Intramuscular priming and intranasal boosting induce strong genital immunity through secretory IgA in minipigs infected with Chlamydia trachomatis. Front Immunol. 2015; 6: 628 10.3389/fimmu.2015.00628 26734002 PMC4679855

[18] Bracho G, Lastre M, del Campo J. Proteoliposome derived cochleate as novel adjuvant. Vaccine. 2006; 24(S2): 30–31. 10.1016/j.vaccine.2005.01.108 16823914

[19] Rodriguez T, Pérez O, Ugrinovic S, Bracho G, Mastroeni P. Bacterial derived proteoliposome as ideal delivery system and cellular adjuvant. Vaccine. 2006; 24(S2): 24–25. 10.1016/j.vaccine.2005.01.106 16823912

[20] Rodriguez T, Pérez O, Ménager N, Ugrinovic S, Bracho G, Mastroeni P. Interactions of proteoliposomes from serogroup B Neisseria meningitidis with bone marrow-derived dendritic cells and macrophages: adjuvant effects and antigen delivery. Vaccine. 2005; 23: 1312–1321. 10.1016/j.vaccine.2004.07.049 15652674

[21] Pérez O, Cabrera O, del Campo J. New vaccines require potent adjuvants like AFPL1 and AFCo1. Scand J Immunol. 2007; 66: 271–277. 10.1111/j.1365-3083.2007.01981.x 17635804

[22] del Campo J, Zayas C, Romeu B. Mucosal immunization using proteoliposome and cochleate structures from Neisseria meningitidis serogroup B induce mucosal and systemic responses. Methods. 2009; 49(4): 301–308. 10.1016/j.ymeth.2009.03.025 19410000

[23] del Campo J, Lindqvist M, Cuello M. Intranasal immunization with a proteoliposome-derived cochleate containing recombinant gD protein confers protective immunity against genital herpes in mice. Vaccine. 2010; 28: 1193–1200. 10.1016/j.vaccine.2009.11.035 19945418

[24] Pérez O, Bracho G, Lastre M. Novel adjuvant based on a proteoliposome-derived chochleate structure containing native lipopolysaccharide as a pathogen-associated molecular pattern. Immunol Cell Biol. 2004; 82: 603–610. 10.1111/j.1440-1711.2004.01293.x 15550118

[25] Oliva R, Fariñas M, Infante JF, Hernández T. Local tolerance study of the VA-MENGOC-BC^®^ antimeningococcal vaccine in Sprague Dawley rats. Evaluation at 24 and 36 months of shelf. VacciMonitor. 2019; 28(1): 9–18.

[26] Ferreira, T, Rasband, W. ImagJ User Guide, Version 1.43. National Institutes of Health, USA. 2010. http://rsb.info.nih.gov/IJ

[27] Matta SG, Balfour DJ, Benowitz NL, et al Guidelines on nicotine dose selection for in vivo research. Psychopharamacology. 2007; 190(3)” 269–319. 10.1007/s00213-006-0441-0 16896961

[28] Canadian Council on Animal Care (CCAC) Guide for the care and use of laboratory animals. 8th Edition, 2010. http://www.nap.edu/catalog.php?record_id=12910.

[29] Suckow, MA, Danneman, P, Brayton, C. The laboratory mouse. CRC Press LLC. http://www.med.unlp.edu.ar/archivos/cicual/the_laboratory_mouse__2001_.pdf.

[30] Cerutti A, Chen K, Chorny A. Immunoglobulin responses at the mucosal interface. Annu Rev Immunol. 2011; 29: 273–93. 10.1146/annurev-immunol-031210-101317 21219173 PMC3064559

[31] WHO Technical Report Series No 927. 2003 https://www.who.int/biologicals/technical_report_series/en/

[32] López Y, Infante JF, Sifontes S, Díaz D, Pérez V, Año G, et al Pharmacology and toxicology of an oral tablet whole cells inactivated cholera vaccine in Sprague Dawley rats. Vaccine. 2011; 29(19): 3596–3599. 10.1016/j.vaccine.2011.02.074 21385634

[33] Baek YO, Choi SK, Shin SH, Koo KH, Choi HY, Cho SB, et al A 6-Week Oral Toxicity Study of Oral Cholera Vaccine in Sprague-Dawley Rats. Toxicological Research. 2012; 28(4): 225–233. 10.5487/TR.2012.28.4.225 24278614 PMC3834433

[34] Fariñas, M, Oliva, R, Infante JF, Valdez, Y, Nuñez, D, Valmaceda, T. Ensayo piloto de inmunogenicidad y toxicidad preclínica de la vacuna Salmonella typhi conjugada en ratas Sprague Dawley. Retel No. 44. 2014. http://www.sertox.com.ar/modules.php?name=Content&pa=showpage&pid=936.

[35] Hamilton JL. Evaluation of fever in infants and young children. Am Fam Physician. 2013; 87(4): 254–260. 23418797

[36] Fujihashi K, Koga T, van Ginkel FW, Hagiwara Y, McGhee JR. A dilemma for mucosal vaccination: efficacy versus toxicity using entertoxin-based adjuvants. Vaccine. 2002; 20(19–20): 2431–38. 10.1016/s0264-410x(02)00155-x 12057597

[37] Chakravarty S, Herkenham M. Toll-like receptor 4 on nonhematopoietic cells sustains CNS inflammation during endotoxemia, independent of systemic cytokines. J Neurosci. 2005; 25(7): 1788–96. 10.1523/JNEUROSCI.4268-04.2005 15716415 PMC6725921

